# Calreticulin in renal fibrosis: A short review

**DOI:** 10.1111/jcmm.17627

**Published:** 2022-11-28

**Authors:** Panagiotis K. Politis, Aristidis S. Charonis

**Affiliations:** ^1^ Center for Basic Research Biomedical Research Foundation of the Academy of Athens Athens Greece; ^2^ Center for Clinical, Experimental Surgery and Translational Research Biomedical Research Foundation of the Academy of Athens Athens Greece; ^3^ University Research Institute of Maternal and Child Health and Precision Medicine Athens Greece

**Keywords:** Calreticulin, chronic kidney disease, mouse models, proteomics

## Abstract

Fibrosis is a common denominator of several pathological conditions. Over the last decade, Calreticulin has emerged as a critical player in the fibrotic processes in many tissues and organs. Here we review the recent advances in our understanding of the regulatory roles of Calreticulin in renal fibrosis. In particular, a proteomic screen that we performed more than 15 years ago, for the identification of novel components involved in the mechanisms of renal fibrosis, led to the observation that Calreticulin is associated with the initiation and progression of kidney fibrosis in a rodent model. We also showed that altered expression levels of Calreticulin in vitro and in vivo are significantly affecting the fibrotic phenotype in cellular systems and animal models, respectively. We also identified an upstream regulatory mechanism that mediates the transcriptional control of Calreticulin expression during the progression of renal fibrosis, by showing that the druggable orphan nuclear receptor NR5A2 and its SUMOylation is involved in this action. These data provide novel targets for future pharmacological interventions against fibrosis. In addition, further proteomic analysis uncovered a correlation between the up‐regulation of Calreticulin and that of 14‐3‐3σ protein. Collectively, our previous observations suggest that Calreticulin is a central node in a regulatory axis that controls the initiation and progression of renal fibrosis.

## 1 FIBROSIS IN GENERAL

Fibrosis is defined as the accumulation of excessive amounts of extracellular matrix in tissues and organs. This excess is the result of either increased production, decreased degradation or a combination of these two processes. In fibrosis, the produced matrix is not only pathological in a quantitative manner, but it is also pathological in qualitative terms since the produced matrix is usually different from the one existing physiologically.^1^ Fibrosis is a pathological process that severely compromises the quality of life and eventually leads to higher mortality risk. It is estimated that 45% of all deaths worldwide have as background the fibrotic phenomenon.^2^

Work from several laboratories has revealed the multi‐facet process of the development of fibrosis: several cell types are involved, there is cross‐talk between resident cells of an organ and specialized cells of the immune system, different cell types may be activated and change their phenotype (from epithelial to mesenchymal, from endothelial to mesenchymal, etc.) and the secretory profile of cells may be drastically altered, following changes in signalling pathways.^3^, ^4^, ^5^, ^6^

Therefore, it is of great importance to better understand the phenomenon, the molecular elements and networks involved as well as the organ‐specific differences in the fibrotic process. This knowledge will lead to novel insights into the development of more effective and targeted therapeutic interventions. Along those lines, this short review summarizes in a concise way the work carried on in the laboratory of the authors by which calreticulin was shown to be involved in the process of renal fibrosis, the mechanism of its involvement was uncovered and several targets for future therapeutic interventions were also revealed.

## 2 RENAL STRUCTURE AND FUNCTIONS

The kidneys are important organs, absolutely essential for life and homeostasis, performing critical functions: First and foremost, they are involved in the metabolism, secretion and reabsorption of ions and small molecules and regulating acid–base equilibrium of body fluids. Despite their small size (they weigh 1/200 of the body weight), they receive one‐fourth of the cardiac output. Every day, they receive about 1800 L of blood, and they produce about 1 L of urine under physiological conditions. Second, they are important regulators of vascular pressure. Third, they are parts of the endocrine system, producing hormones such as renin and erythropoietin and are the target of several hormones, such as aldosterone, anti‐diuretic hormone and natriuretic peptide.

Anatomically, they are bean‐shaped, having a peripheral cortex and a centrally located medulla. A light microscopic view uncovers lots of tubules (of different sizes of epithelial cells) and globular structures, the glomeruli, where vessels exchange their content with the beginning of the tubular system.

## 3 EFFECT OF FIBROSIS ON RENAL STRUCTURE AND FUNCTION

Fibrosis affects the two major compartments of the renal parenchyma. It leads to the thickening of the glomerular basement membrane and expansion of the mesangial matrix in the glomeruli and thickening of the tubular basement membranes (Liu et al, 2011^7^;). This process compromises the function of the kidneys and leads to what is known as chronic kidney disease (CKD). CKD is defined physiologically as the reduction in the ability of the kidneys to perform their function, that is the reduction of the glomerular filtration rate (GFR) from 120 ml/min to lower values. The reduced values correspond to different stages of CKD, the last one, stage 5 (where GFR is lower than 15) being incompatible with life and requiring replacement therapies (either transplantation or dialysis).

CKD is a very common condition. It has recently been realized from independent studies in North America, Europe and Asia that about 10%–13% of the adult population suffers from CKD at a certain stage (1–5).^8^, ^9^, ^10^

Based on how common CKD is and how much it affects the health of the population, especially the ageing population, it is important first, to develop novel biomarkers for the detection of renal fibrosis at the early stages of its development and second, to understand the molecular level of the underlying mechanisms. This understanding will uncover specific targets for therapeutic interventions depending on the aetiology, the cell type(s) involved and the stage of the pathological process.

## 4 PROTEOMIC APPROACH TO RENAL FIBROSIS: CALRETICULIN IDENTIFICATION IN THE UUO MODEL

To fulfil these two aims, our group has used rodent models for renal fibrosis. For the studies of renal fibrosis, several animal models have been used, mainly in rodents. These can be classified as induced, spontaneous and genetic.^11^ Induced models can be generated after administration of toxic chemicals (adriamycin, folic acid, streptozotocin, cyclosporine, mercuric chloride, vanadate and others), radiation or surgical intervention. There are three models of renal fibrosis following surgical intervention: Ischaemia–reperfusion, mass reduction (also known as 5/6 nephrectomy) and Unilateral Ureteric obstruction (UUO). Among all these models, we have selected to use in our studies the UUO model for a number of reasons: it has been applied successfully to both rats and mice of all strains; it produces full‐blown fibrosis in a very short time interval (1–2 weeks); the same animal carries the control sample of renal changes in the healthy kidney; the pathological changes are similar to the changes observed in human fibrosis both in the glomerular and in the tubular compartment of the kidney. For all these reasons, the UUO model is the most widely used model so far and therefore we decided to use this model in our studies. In this model, obstruction of one ureter is compatible with life but the animal develops fibrosis in a matter of few days in the corresponding (ipsilateral) kidney, with no sign of fibrosis in the contralateral kidney.

Since fibrosis is a rather complicated and multifactorial process, we decided to apply system biology approaches, more specifically proteomics. Proteomics can be an ideal approach for complicated biological processes since their use can uncover macromolecules that are involved in these processes that were not previously identified and suggest the involvement of new biochemical pathways.^12^, ^13^

Using kidney samples taken at various time intervals following ureteric ligation, we analysed them in 2D gel electrophoresis and then identified several proteins that are up‐regulated or down‐regulated during the development of fibrosis. Among them, calreticulin was one that was consistently up‐regulated from early time intervals and in all animals examined.^14^ This protein up‐regulation was due to transcriptional control since the expression of calreticulin mRNA was also up‐regulated in a time‐dependent manner. These data were reflecting a biochemical change in the renal parenchyma; however, it was also necessary to define where, that is which renal compartment or cell type, was responsible for the observed up‐regulation. To this end, immunohistochemistry and immunofluorescence approaches indicated that the up‐regulation was by far more pronounced in the tubular epithelial cells.^14^

## 5 MECHANISM OF UP‐REGULATION OF CALRETICULIN EXPRESSION

**FIGURE 1:**
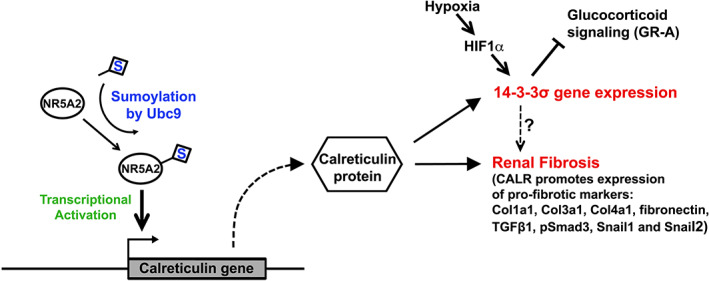
Schematic representation of our proposed model of the involvement of Calreticulin in renal fibrosis. A detailed description of the upstream and downstream factors that we have recently identified to be associated with Calreticulin function, is provided in the main text. CALR, Calreticulin; NR5A2, Nuclear Receptor 5A2; Ubc9, also known as Ube2I (ubiquitin‐conjugating enzyme E2 I); HIF1α, Hypoxia Inducible Factor 1 subunit alpha.

Our next aim was to elucidate the molecular mechanism of calreticulin up‐regulation. To do that, we focused on the promoter of the calreticulin gene. Binding sites for transcription factors known to be involved in fibrosis were considered, along with binding site conservation in several species. A list of 17 candidates transcription regulators was short‐listed and corresponding expression vectors were constructed. Among them, NR5A2 was found to significantly transactivate calreticulin promoter‐luciferase construct in renal cell lines, compared to all other members of the list. In addition, chromatin immunoprecipitation assays in samples from the animal model used showed specific binding of NR5A2 at several sites close to the transcription start site of the calreticulin gene (Figure 1). This binding was associated with the enrichment of RNA Pol II binding and H3K4me3 on the calreticulin promoter. Furthermore, real‐time RT‐qPCR assays confirmed the up‐regulation of NR5A2 during the progression of fibrosis in the UUO model. Immunohistochemistry demonstrated that both the renal cortex and the renal medulla were expressing high levels of NR5A2 in fibrosis and this phenomenon was localized both in the nuclear and the cytoplasmic compartment of tubular epithelial cells.^15^ Western blotting experiments confirmed the up‐regulation of NR5A2 in samples from fibrotic kidneys, however, altered mobilities suggested a major post‐translational modification. Further studies uncovered that SUMOylation was involved in the process, as confirmed by immunoblotting, immunoprecipitation and immunohistochemistry approaches. In addition, a K224R‐mutated expression vector of NR5A2, which makes it unable to be SUMOylated, is also unable to up‐regulate calreticulin in transfection experiments. Since the key enzyme catalysing this post‐translational modification is UBC9, we further studied its expression in our renal fibrosis model by real‐time RT‐qPCR, Western blotting and immunohistochemistry. All approaches confirmed an up‐regulation of UBC9 during the development of fibrosis in renal parenchyma.^15^

In conclusion, activation of UBC9, during the fibrotic process, seems to promote SUMOylation of transcription factor NR5A2 and enhance its ability to activate calreticulin transcription (Figure 1).

## 6 PHENOTYPIC CHANGES DURING DOWN‐REGULATION OF CALRETICULIN EXPRESSION

Following the described mechanistic studies, we decided to evaluate the changes in the fibrotic process when calreticulin is down‐regulated. For this purpose, we decided not to use a knockout animal model, since severe phenotypic changes would affect the model; instead, we decided to use mice heterozygous for calreticulin, provided by Prof. Michalak,^16^ which express half the amount of normally expressed calreticulin. These mice were used as our model of renal fibrosis (UUO).

Initially, we also confirmed that calreticulin expression was reduced to about 50% in the renal tissue of these animals. Macroscopically, the UUO‐induced damage in the kidneys of heterozygous animals was less severe compared with the control (homozygous for Carleticulin) animals. For example, they displayed less severe enlargement and fibrosis‐induced colour change, compared to the kidneys of homozygous animals.

At the light microscopic level, staining with Sirius Red indicated a major reduction in the accumulation of extracellular matrix in the kidneys of heterozygous animals, reaching 50% at the 17‐day interval. Therefore, clear protection against the progression of fibrosis was observed when levels of calreticulin were reduced.^17^ The heterozygous animals also exhibited a reduced expression in several pro‐fibrotic genes examined, such as Col1a1, Col3a1, Col4a1, fibronectin, TGFβ1, phosphor Smad3, Snail1 Snail2 (Figure 1). In addition, E‐cadherin, a marker of epithelial integrity was reduced and α‐SMA‐positive myofibroblasts were increased in control animals compared to heterozygous ones. Similar results were obtained with pro‐inflammatory mediators TNF‐α and MCP1. In addition, renal tubular cells of heterozygous animals were protected from apoptosis compared to controls. We examined also the expression of thrombospondin 1 (TSP1), a molecule playing a critical role in the activation of TGFβ1 and, similarly, its expression levels were reduced in heterozygous animals. TSP1 is a matricellular extracellular matrix protein that has diverse roles in regulating cellular processes important for the pathogenesis of fibrotic diseases, with a major role in renal fibrosis.^18^ These data are all supporting the notion that calreticulin positively regulates pro‐inflammatory and profibrotic pathways and its reduction has beneficial effects on these processes.^17^

## 7 PHENOTYPIC CHANGES DURING UP‐REGULATION OF CALRETICULIN EXPRESSION

In parallel, we studied how the up‐regulation of calreticulin specifically in renal tubular epithelial cells affects the progression of fibrosis. For that purpose, we used the well‐differentiated proximal tubule epithelial cell line HK‐2 that we genetically modified to stably overexpress calreticulin. We have also produced a similar cell line as a control, using the empty vector. Calreticulin overexpressing cells exhibited a different phenotype compared to controls, being more elongated, mesenchymal‐like, adhering more loosely to each other and with increased mobility in wound healing assays. Interestingly, the levels of GRP78, an important endoplasmic reticulum macromolecule involved in stress sensing, were increased, indicating increased ER stress. Also, TUNEL assays indicated an increase in apoptosis; these results were confirmed with immunohistochemistry with caspase‐3, where calreticulin overexpressing cells were exhibiting increased levels of this apoptosis‐specific marker.^17^ These observations prompted us to proceed to a more detailed study of the changes in proximal tubule epithelial cells caused by calreticulin overexpression. Two independent clones of tubular epithelial cells overexpressing calreticulin and two control clones were studied using proteomic analysis and 2D gel electrophoresis (details of the methodology are described in^19^). The results from the proteomic analysis demonstrate alterations in the expression of proteins involved in inflammation, extracellular matrix production, cytoskeleton, metabolism, apoptosis, protein folding and degradation, chaperones, heat shock, cell cycle and RNA splicing.^19^ Among those, of particular interest to us was the family of 14‐3‐3 proteins, since this family was not associated with renal pathology so far. We focused on a specific member of this family of proteins, namely 14‐3‐3σ, also known as stratifin.^20^ We detected up‐regulation of 14‐3‐3σ from early stages in samples from the UUO model at both mRNA and protein levels. In addition, histochemical staining confirmed the up‐regulation of 14‐3‐3σ at the tubular compartment (more pronounced in distal tubules) of UUO kidneys. Interestingly, up‐regulation of the family of 14‐3‐3 proteins was detected in other models (nephrotoxic serum and ischaemia–reperfusion) of renal pathologies. Since in all three models a common characteristic is the presence of hypoxia, we tested the effect of hypoxia on primary cultures of tubular epithelial cells. Thus, we confirmed that hypoxia up‐regulates 14‐3‐3σ expression, and by using chromatin immunoprecipitation assays we showed that HIF1a is directly recruited to the 14‐3‐3σ promoter^19^ (Figure 1).

Studies of the interactome of 14‐3‐3σ reveal a strong association with the family of keratins, which are known to be involved in cytoskeletal dynamics and intracellular signalling.^21^ In particular, Keratins 8 and 18 are subject to phosphorylation by Raf1 kinase, a biochemical event in which the 14‐3‐3 family members play a crucial role.^22^ Therefore, our findings provide a mechanistic explanation for the observation that keratins 8 and 18 are up‐regulated and hyper‐phosphorylated in renal diseases both in animal models and in humans (Djudjai et al, 2016).

Moreover, our data indicated that during the development of fibrosis, the staining for 14‐3‐3σ is mainly cytoplasmic. This is of interest since a previous study suggests that 14‐3‐3σ interacts and favours cytoplasmic localization of glucocorticoid receptor A (GR‐A), thus acting as a disruptor of glucocorticoid signalling^23^ (Figure 1). This is of importance since glucocorticoid signalling affects many pathways and regulates numerous gene expression programs, implying that calreticulin up‐regulation may have many yet undiscovered effects.

One of the most common renal pathologies is diabetic nephropathy. In this case, renal fibrosis in humans develops gradually, over many years. In a recently published elegant study, both in vitro and in vivo approaches were used to evaluate the role of calreticulin in fibrosis in diabetic nephropathy. Calreticulin was up‐regulated when cultured renal tubular cells were stimulated with high glucose or TGF‐β; the knockdown of calreticulin by siRNA led to reduced production of collagen. Calreticulin was also increased in diabetic mice; again, the knockdown of calreticulin expression improved the morphology and the function of the kidneys.^24^

## 8 STUDIES IN HUMAN TISSUES

All the above studies were performed in tissues taken from rodent models of nephropathy. In order to explore the possibility that altered expression is also occurring in human renal pathologies, we performed immunohistochemical studies in renal sections from patients suffering from IgA nephropathy and membranous nephropathy and compared them with renal sections from control materials (kidneys removed due to surgery). Similar to the rodent and cellular models, calreticulin (and 14‐3‐3σ) were up‐regulated in all patient sections examined in both pathological conditions. This up‐regulation was prominent in the tubular epithelial cells and reached the highest level in distal tubule epithelial cells.^19^

## 9 POSSIBLE PHARMACOLOGICAL INTERVENTIONS

Based on the findings summarized above, we propose that future pharmacological interventions in order to reduce the expression of calreticulin might be worth examining.

Two different approaches could be envisioned: first, it would be interesting to test the effect of reducing the translation of calreticulin mRNA by using specific anti‐sense oligonucleotides. This is a method described in the past and specific modifications to make the oligos more effective have been proposed.^25^ The beneficial effect of the application of therapeutic anti‐sense oligonucleotides has been documented in several animal models with renal pathologies.^26^, ^27^, ^28^

Second, it would be of interest to evaluate the effect of applying antagonists of NR5A2 on the progress of renal fibrosis. Such molecules do exist and have already been studied in different contexts.^29^, ^30^

In addition, NR5A2 can interact with the phospholipid dilauryl‐phosphatidyl choline and this interaction may have anti‐diabetic, anti‐tumorigenic and lipotropic effects.^31^, ^32^ Along the same lines, NR5A2 has been suggested as a crucial element in the pathway of endoplasmic reticulum stress resolution in the liver (Marmosh et al, 2014). In this context, NR5A2 has been proposed to activate polo‐like kinase 3 (PLK3), which phosphorylates Activating Transcription Factor 2 (ATF2). In our fibrosis model (UUO), we have observed a higher than four‐fold up‐regulation of PLK3. Therefore, it should be kept in mind that NR5A2 may have important other functions in parallel with the up‐regulation of calreticulin gene transcription.

## 10 CALRETICULIN IN FIBROSIS OF OTHER ORGANS

There are few interesting reports about the role of calreticulin in the fibrotic processes in other organs: in bone marrow,^33^ in the lung (Zang et al, 2017), in the heart.^34^ A lot needs to be learned regarding mechanistic differences and common pathways induced by calreticulin in a tissue‐dependent manner regarding all these organs including the kidneys and liver.

## AUTHOR CONTRIBUTIONS

**Panagiotis Politis:** Writing – review and editing (equal). **Aristidis S. Charonis:** Conceptualization (equal); supervision (equal); visualization (equal); writing – original draft (equal); writing – review and editing (equal).

## CONFLICT OF INTEREST

The authors declare that they have no conflict of interest.

## Data Availability

The data that support the findings of this study are openly available
